# Exome Sequencing in Pacific Islanders With Nephropathies of Unknown Origin

**DOI:** 10.1016/j.ekir.2025.03.005

**Published:** 2025-03-10

**Authors:** David de Saint Gilles, Nicolas Quirin, Yannis Lombardi, Thomas Lamy, Yann Butté, Antoine Biron, Marine Dancer, Laure Raymond, Laurent Mesnard, Alice Doreille

**Affiliations:** 1 Soins Intensifs Néphrologiques et Rein Aigu, Hôpital Tenon, Assistance Publique-Hôpitaux de Paris, Paris, France; 2 Service de néphrologie, Centre Hospitalier Territorial, Medipole, Dumbéa sur mer, Nouvelle-Calédonie; 3 Faculté de médecine, Sorbonne Université, Paris, France; 4 Département de biologie, Medipole, Dumbéa sur mer, Nouvelle-Calédonie; 5 Département de génétique, Eurofins-Biomnis, Lyon, France; 6 INSERM UMR 1155, Hôpital Tenon, Paris, France; 7 Service de Néphrologie, Centre Hospitalier de l’université de Montréal, Québec, Canada

**Keywords:** chronic kidney disease, genetics, reference genome

## Introduction

New Caledonia and Wallis and Futuna Islands are French overseas collectivities. The incidence of end-stage kidney disease is 2.4 times greater than in mainland France,^1^ with young patients (55% of dialyzed patients aged < 65 years). Moreover, nephropathy of unknown origin represents 47% of the diagnoses in these islands. Given the insular nature of the population and emerging data in adult nephrology on the value of genetic sequencing in undetermined nephropathies,^2^^,^^3^ we have begun evaluating genetic investigations in this population.

From November 2019 to January 2022, in the nephrological care structures in New Caledonia and Wallis and Futuna, we used exome sequencing (ES) as the first-tier genetic exploration of adult nephropathy of unknown origin when red flags suggestive of genetic kidney disease were present (family history of end-stage kidney disease, consanguinity, syndromic presentation, and early onset of chronic kidney disease [CKD]). The referring nephrologist completed an ES order, specifying demographic and clinical data, and obtained consent for genetic analysis. All the coding regions of the genome (RefSeq exons) were sequenced on Illumina platforms, and analyzed using dedicated software (SeqOne, Montpellier, France). Variant segregation was explored using targeted approaches in relatives. Copy number variant analysis was performed from exome assay and based on GATK4. All patients’ sequencing data were discussed during a multidisciplinary genome board meeting, which included at least a laboratory geneticist and a nephrologist with a special interest in nephrogenomics, and the local nephrologist who ordered the ES for the patient under discussion. Diagnostic variants were “pathogenic” or “likely pathogenic” according to the American College of Medical Genetics and Genomics guidelines explicative of the patient’s phenotype.^4^

The study was performed in accordance with the ethical standards of the Declaration of Helsinki. Written informed consent was obtained from all patients regarding de-identified clinical and personal patient data collection, analysis, and publication.

## Results

Since November 2019, a total of 88 unrelated patients underwent ES to explore their nephropathy. Patients’ baseline characteristics are presented in Table 1.

**Table 1:** Population description

Population	Overall	ES+	VUS+
Total exome sequencing, *n* (%)	88 (100)	14 (16)	33 (38)
Age at ES, median [IQR]	44 [37–54]	40 [37–49]	46 [40–54]
Sex (females), *n* (%)	45 (51)	4 (29)	22 (67)
Ethnicity, *n* (%)			
Melanesian	52 (59)	10 (71)	22 (67)
Polynesian	27 (31)	1 (7)	9 (27)
Caucasian	7 (8)	2 (14)	2 (6)
Asian	3 (3)	1 (7)	0
Other	1 (1)	0	0
CKD stage at time of ES, *n* (%)			
I	6 (7)	2 (14)	0
II	3 (3)	2 (14)	1 (3)
III	9 (10)	1 (7)	1 (3)
IV	8 (9)	0	3 (9)
Total stage V (including dialyzed and transplanted patients)	62 (70)	9 (64)	28 (85)
Familial history, *n* (%)			
First degree family history of nephropathy	51 (59)	9 (64)	22 (67)
Second degree family history of nephropathy	46 (52)	5 (36)	18 (55)
Consanguinity	5 (6)	1 (7)	3 (9)
Early onset CKD with familial nephropathy	27 (31)	4 (29)	9 (27)
Diagnoses before ES, *n* (%)			
Glomerulopathy	21 (24)	3 (21)	7 (21)
Vascular nephropathy	26 (30)	3 (21)	9 (27)
Diabetic nephropathy	15 (17)	1 (7)	8 (24)
Unclassified disease	17 (19)	4 (29)	8 (24)
Tubulo-interstitial nephropathy	8 (9)	1 (7)	2 (6)
Isolated proteinuria or hematuria	2 (2)	1 (7)	0
Cystic or developmental nephropathy	5 (6)	1 (7)	2 (6)
Other	4 (5)	1 (7)	0
Factors motivating ES			
Nephropathy of unknown origin	70 (80)	11 (79)	27 (71)
Early CKD onset (aged < 35 yrs)	53 (60)	9 (64)	18 (55)
First and/or second-degree nephropathy	68 (77)	9 (64)	28 (85)

CKD, chronic kidney disease; ES, exome sequencing; IQR, interquartile range; VUS, variant of undetermined significance.

Mean age was 44 (37–54) years and 45% of patients were women. Self-declared ethnic ancestry was 59% Melanesian, 31% Polynesian, 8% White European, 3% Asian, 1% other. A first-degree family history of CKD was found in 59% of cases.

We detected diagnostic variants in 14 of 88 patients (diagnostic yield: 16%) (in Table 2). Among the 14 patients with a molecular diagnosis, 11 were of Oceanian origin.

**Table 2:** Molecular and clinical characteristics of patients with positive exome

ID	Gene(s)	Zygosity	NM	c.HVGS	p.HgVS	ACMG classification	Type of variation	Clinical phenotype	Origin	Cascade screening
1	*COL4A4*	Homozygous	NM_000092.4	c.2752G>A	p.Gly918Arg	LP- PM1, PM2, PP2, PP3	Missense	ESKD at age 37 yrs, nephropathy of unknown origin, family history of CKD (*n* = 3), no other clinical phenotype for specific orientation. No KB.	Melanesian	4 relatives tested: 2 with CKD carrying the variant, 2 potential kidney donors not carrying the variant
8	*UMOD*	Heterozygous	NM_003361.3	c.481T>C	p.Cys161Arg	LP- PM2, PP2, PP3	Missense	ESKD at age 52 yrs, hypertensive nephropathy, family history of CKD (*n* = 3), no other clinical phenotype for specific orientation. No KB.	Melanesian	5 relatives tested: 3 with CKD carrying the variant, 2 potential kidney donors not carrying the variant
35	*COL4A4*	Heterozygous	NM_000092.5	c.2752G>A	p.Gly918Arg	LP- PM1, PM2, PP3, (PP5)	Missense	Isolated proteinuria/hematuria, family history of CKD (*n =* 1). KB: basement membrane abnormality	Melanesian	No
36	*CLCN5*	Hemizygous	NM_000084.3	c.206-1G>C	NA	LP- PVS1, PM2, (PP5)	Splice variant	ESKD at age 49 yrs, nephropathy of unknown origin, family history of CKD (*n =* 3). Gout at age 30 yrs. No KB.	Melanesian	1 healthy relative not carrying the variant
44	*COL4A4* *GUCY1A3*	Heterozygous Homozygous	NM_000092.5 NM_000856.5	c.1706G>C c.5del	p.Gly569Alap.Phe2SerfsTer8NA	LP–PM1, PM2, PP2, PP3 -	Missense LOF	ESKD at age 28 yrs, hypertensive nephropathy, no family history of CKD. Achalasia. KB: acute vascular hypertension	Melanesian	No
59	*UMOD*	Heterozygous	NM_003361.3	c.667T>G	p.Cys223Gly	LP - PM1, PM2, PM5, PP2, PP3	Missense	CKD before 35 yrs, family history of CKD (*n =* 2). Dysthyroidism and hypertension, gout at age 35 yrs. KB: tubulointerstitial lesions.	White European	2 relatives tested: 1 healthy sister not carrying, 1 dialyzed sister carrying the variant
70	*ELN*	Heterozygous	NM_001278939.2	dup(7) (q11.23q11.23) chr7:g.73303128_74757131dup	NA	1A 3 A 4C 4 L	Duplication (1.4Mb)	ESKD at age 38 yrs, nephropathy of unknown origin. No KB. No family history. Moderate mental retardation. Consanguinity. No KB.	Melanesian	No
74	*COL4A4*	Compound Heterozygous	NM_000092.5	c.2752G>A c.1051C>T	p.Gly918Argp.His351Tyr	LP- PM1, PM2, PP2, PP3	Missense Missense	ESKD at age 36 yrs, nephropathy of unknown origin, family history of CKD (*n =* 2). No KB.	Melanesian	No
77	*CFI*	Heterozygous	NM_000204.3	c.1621_1623delinsACATTGTGAGTT	p.Trp541_Val583delinsThrLeu	LP- PM1, PM2, PM4, PP3	Delins	ESKD at 30 yrs, for unknown nephropathy. Hypertension with TMA. C3 low. No KB	Melanesian	No
79	*COL4A4*	Heterozygous	NM_000092.5	c.2752G>A	p.Gly918Arg	LP- PM1, PM2, PP2, PP3	Missense	CKD 46 yrs, family history of CKD (*n =* 3). No KB.	Asian	Offered to 3 relatives but not performed yet
84	*PAX2*	Heterozygous	NM_003987.5	c.226G>A	p.Gly76Ser	LP- PM1, PM2, PM5, PP2, PP3	Missense	ESKD at age 25 yrs, family history of CKD (*n =* 2). Cystic kidneys. No KB	Melanesian	Offered to relatives but not performed yet
95	*MT-TL1* *TG*	Heteroplasmic Homozygous	Mitochondrial^a^ NM_003235.5	m.3243A>G c.886C>T	NA p.Arg296Ter	P: PS1 PS3 PS4 PM1 PM2 PM5 PP3 P–PVS1, PM2, PM3, (PP5)	Missense Nonsense	CKD before 35 yrs. Congenital hypothyroidism. Hypertrophic cardiomyopathy. Deafness. Diabetes. No family history of CKD. Epilepsy in mother and sister. KB: IFTA	White European	No
99	*SCN4A*	Heterozygous	NM_000334.4	c.4786A>G	p.Ile1596Val	LP–PM1, PM2, PP2, PP4	Missense	Acute hypokalemia with tetraparesis. No KB. No family history.	Polynesian	No
109	*PAX2*	Heterozygous	NM_003990.4	chr10 :g.102539100_102547419del	NA	1A, 2A-2E, 3A	Deletion	ESKD at age 9 yrs, bilateral renal hypoplasia. No family history. No KB	Melanesian	No

ACMG, American College of Medical Genetics and Genomics; CKD, chronic kidney disease; CNV, copy number variant; ESKD, end-stage kidney disease; IFTA, interstitial fibrosis and tubular atrophy; KB, kidney biopsy; LOF, loss of function; LP, likely pathogenic; P, pathogenic; NA, not applicable; TMA, thrombotic microangiopathy.

a 40% of heteroplasmy in blood.

A total of 33 patients (38%) presented a variant of unknown significance “+” (VUS+, i.e., variants estimated to lie close to the border between uncertain significance and likely pathogenic) related to the phenotype.

Among the 33 VUS+ identified (Supplementary Table S1), 10 were in *HNF1B*, 3 in *COL4A4*, and 4 in *COL4A5*.

Among the 10 patients with a VUS+ in *HNF1B*, 8 had at least 1 family member with a history of CKD. Their clinical phenotypes were particularly heterogeneous, with 5 patients presenting with diabetes and 5 developing CKD before the age of 35 years. The prevalence of *HNF1B*:c.780G > C and c.1654-4G > A variants was 5.7% in our cohort, whereas they were rare in GnomAD (0.0078% and 0.0004%, respectively). Among the 7 patients with a VUS+ in *COL4A5* and *COL4A4*, 6 reported a first-degree familial nephropathy, 6 had reached end-stage kidney disease. For 4 patients, the initial diagnosis was a tubulointerstitial or vascular nephropathy; for 1 patient, a malformative nephropathy; and for 2 patients, a nephropathy of unknown origin.

No *APOL1* risk alleles were found in the 88 patients of the cohort, likely because of an absence of selection pressure from Trypanosomiasis in Oceania.

A total of 3 patients had genetic findings related to a non kidney feature matching their clinical phenotype: *KCNH* (long QT syndrome), *TG* (thyroid dyshormonogenesis), and *GUCY1A3* (achalasia, Moyamoya syndrome).

In multivariate analysis, including sex, age, ethnicity, family history of CKD, consanguinity and clinical presentation, no factor was significantly associated with obtaining a molecular diagnosis.

## Discussion

This cohort study demonstrated a diagnostic yield of 16% in a population of adult nephrology patients in New Caledonia. Even though lower than the yield observed in Europe and the USA,^2^^,^^3^ establishing a molecular diagnosis has major implications.

For example, cascade screening by targeted gene sequencing led to multiple diagnoses in populations often with numerous families for a modest cost (130€ for a Sanger sequencing). Second, it enabled living donor kidney transplantation. Indeed, the first-degree cousin of an index case also had end-stage kidney disease and her younger sister was willing to donate, but the medical team declined the proposal given the family history. After identifying the pathogenic variant in the index case, the potential donor's eligibility for living donation could be established by confirming the absence of this mutation in the donor. Finally, the identification of a genetic cause of nephropathy prevented nephrologists from performing kidney biopsies in relatives of index cases in at least 1 identified case. A kidney biopsy in New Caledonia means 3 days hospitalization (2900€) and shipment of the biopsy specimen to Australia for analysis (additional cost 500€). After the first year, kidney transplantation allows a saving of approximately 60,000€/yr compared with dialysis (local data). The cost of ES (i.e., 1200€) has to be balanced with the savings made possible by the molecular diagnosis. Therefore, establishing molecular diagnoses can potentially improve quality of life in patients and be cost saving if more living donor kidney transplants are performed and kidney biopsies are avoided.

Although our study population is drawn from the entire cohort of patients with CKD in a territory including New Caledonia, Wallis and Futuna, and the Loyalty Islands; the sequenced population is highly selected (nephropathy of unknown origin with red flag suggestive of genetic kidney disease). Consequently, the generalizability of our results to the broader population of patients with CKD across Oceania is limited. The lower yield of ES in New Caledonia patients with CKD might be explained by the difficulty in characterizing variants in this population by their absence in the population’s genetic data, resulting in a high number of VUS. Indeed, Oceania people are underrepresented in genetic studies. Genetic data obtained by ES are compared with a reference genome to identify variations. Although the reference genome is a standard, it is not universal. The allelic diversity within the reference genome is not an average of the global population. In fact, 70% of the first reference genome was obtained from a single individual from Buffalo.^5^^,^^6^ Projects such as 1000 Genome Project, and more recently Genomic England and the Pangenome project, are broadening population variability in genomics data. No samples from Melanesians or Polynesians are registered in the 1000 Genome Project database. In the GnomAD database, widely used to characterize variants, no mention is made of Oceanian people. If data are available about Oceanian people, they might be gathered in the section “Other,” which represents < 2% of the data available in this database. Moreover, only 0.3% of genome-wide association studies included patients of Oceanian origin.^7^ This underrepresentation of Oceanian people might explain the lower diagnostic yield and high number of VUS. This applies to the occurrence of *HNF1B* variants within our sequenced population. *HNF1B* variants have variable penetrance, with the classical phenotype, including CKD, gout, and diabetes. Given the high incidence of CKD, diabetes,^8^ and gout^9^ in Oceanian populations, further investigation is necessary to ascertain whether these variants represent local polymorphisms or genuine pathogenic mutations that could contribute to the local epidemiology.

## Conclusion

Our results argue for more consistent implementation of ES in Oceanian patients with CKD of unknown origin. Frequent VUS variants, such as *HNF1B*, in this population might explain the over incidence of CKD and diabetes in this population, but need further investigation with functional and experimental data. Furthermore, we need to bolster genomic sequencing campaigns dedicated to healthy Oceanian subjects to gain better insights into their genome variability.

## Disclosure

LM declares lecture fees with Travere Pharmaceutical and travel grant with Sanofi France Pharma. LR and MD are employed by Eurofins Biomnis, a private laboratory doing genetic analysis. All the other authors declared no competing interests.

## Supplementary Material

Supplementary File (PDF) **Table S1.** Molecular and clinical characteristics of patients with variants of unknown significance.

## Footnotes

Supplementary File (PDF)
**Table S1.** Molecular and clinical characteristics of patients with variants of unknown significance.
