# Supplementary material for: Exome Sequencing in Pacific Islanders With Nephropathies of Unknown Origin
Source: Kidney Int Rep. 2025 Mar 10;10(6):2054–7. doi: 10.1016/j.ekir.2025.03.005 (PMC12230993; doi:10.1016/j.ekir.2025.03.005)
Supplement: Supplementary File (PDF) — Table S1. Molecular and clinical characteristics of patients with variants of unknown significance. [file mmc1.pdf]

ID	Gene(s)	c.HVGS	NM	Type of variation	Status	Clinical phenotype	Origin
34	*HNF1B*	c.780G>C	NM_000458.2	Missense	Heterozygous	Family history of CKD, ESKD at 55 y/o	Polynesian
40	*HNF1B*	c.1654-4G>A	NM_000458.2	Splice variant	Heterozygous	Family history of CKD, ESKD at 43 y/o	Melanesian
49	*HNF1B*	c.780G>C	NM_000458.2	Missense	Heterozygous	Family history of CKD, ESKD at 56 y/o	Polynesian
50	*HNF1B*	c.1654-4G>A	NM_000458.2	Splice variant	Heterozygous	Family history of CKD, ESKD at 45 y/o	Polynesian
61	*HNF1B*	c.780G>C	NM_000458.2	Missense	Heterozygous	Family history of CKD, nephropathy of unknown origin, ESKD at 34 y/o	Polynesian
77	*HNF1B*	c.780G>C	NM_000458.2	Missense	Heterozygous	Nephropathy of unknown origin, ESKD at 30 y/o, **had a pathogenic variant of CFI (class IV) associated**	Melanesian
78	*HNF1B*	c.780G>C	NM_000458.2	Missense	Heterozygous	Family history of CKD, early CKD, nephropathy of unknown origin	Melanesian
91	*HNF1B*	c.1654-4G>A	NM_000458.2	Splice variant	Heterozygous	nephropathy of unknown origin	Polynesian
107	*HNF1B*	c.1654-4G>A	NM_000458.2	Splice variant	Heterozygous	Familial and early CKD, nephropathy of unknown origin, ESKD at 38 y/o	Melanesian
108	*HNF1B*	c.1654-4G>A	NM_000458.2	Splice variant	Heterozygous	Nephropathy of unknown origin, ESKD at 36y/o	Oceanian
13	*COL4A5*	c.3830A>G	NM_000495.4	Missense	Heterozygous	Family history of CKD, nephropathy of unknown origin, ESKD at 48 y/o	Melanesian
62	*COL4A5*	c.2365A>T	NM_000495.4	Missense	Hemizygous	Family history of CKD, nephropathy of unknown origin, ESKD at 44y/o	Polynesian
67	*COL4A5*	c.2365A>T	NM_000495.4	Missense	Heterozygous	Family history of CKD, nephropathy of unknown origin, ESKD at 46 y/o	Polynesian
90	*COL4A5*	c.4775C>T	NM_000495.4	Missense	Hemizygous	Family history of CKD, nephropathy of unknown origin, early onset CKD	Polynesian
14	*COL4A4*	c.5049C>A	NM_000092.4	Missense	Heterozygous	Familial CKD, nephropathy of unknown origin, ESKD at 42 y/o	Melanesian
75	*COL4A4*	c.4334-3del	NM_000092.4	Splice variant	Homozygous	Family history of CKD, nephropathy of unknown origin, ESKD at 42y/o	Melanesian
98	*COL4A4*	c.244C>T	NM_000092.4	Missense	Heterozygous	Nephropathy of unknown origin, ESKD at 27 y/o	Melanesian
31	*ABCC6*	c.484A>G	NM_001171.5	Missense	Heterozygous	Nephropathy of unknown origin, ESKD at 25 y/o,	Melanesian
45	*ABCC6*	c.2782G>A<br>c.392A>G	NM_001171.5<br>NM_001171.5	Missense<br>Missense	Heterozygous<br>Heterozygous	Family history of CKD, nephropathy of unknown origin, ESKD at 48 y/o	Melanesian
11	*ABCC6*<br>*WT1*	c.2782G>A<br>c.806G>A	NM_001171.5<br>NM_000378.4	Missense<br>Missense	Heterozygous<br>Heterozygous	Family history of CKD, nephropathy of unknown origin, ESKD at 46 y/o	Melanesian
28	*ABCC6*<br>*WFS1*	c.2125G>A<br>c.1520T>C	NM_001171.5<br>NM_006005.3	Missense<br>Missense	Homozygous<br>Heterozygous	Family history of CKD, nephropathy of unknown origin, ESKD at 42 y/o	Melanesian
101	*CHD7*	c.4813G>C	NM_017780.4	Missense	Heterozygous	Nephropathy of unknown origin, ESKD at 24 y/o	Melanesian
27	*SIX 2*	c.363C>G	NM_016932.4	Missense	Homozygous	Family history of CKD, nephropathy of unknown origin, ESKD at 5 y/o	Melanesian
63	*WT1*<br>*FGFR3*	c.895T>A<br>c.2389del	NM_000378.4<br>NM_000142.4	Missense<br>frameshift	Heterozygous<br>Heterozygous	Early onset CKD, nephropathy of unknown origin	Caucasian

48	*ROBO2*<br>*IFIH1*	c.3938C>G<br>c.2044+1del	NM_001290040.1<br>NM_022168.3	Missense<br>Splice variant	Heterozygous<br>Heterozygous	Family history of CKD, nephropathy of unknown origin, ESKD at 58 y/o	Caucasian
106	*EYA1*	c.990T>G	NM_000503.6	Missense	Heterozygous	Family history of CKD, nephropathy of unknown origin	Melanesian
41	*PKHD1*	c.9236C>T<br>c.7726A>G	NM_138694.3<br>NM_138694.3	Missense<br>Missense	Heterozygous<br>Heterozygous	Family history of CKD, nephropathy of unknown origin	Melansian
69	*LCAT*	c.544C>T	NM_000229.1	Missense	Heterozygous	Family history of CKD, nephropathy of unknown origin, ESKD at 40 y/o	Melanesian
87	*AHI1*	c.1516C>T	NM_017651.4	Missense	Heterozygous	Family history of CKD, nephropathy of unknown origin, ESKD at 29 y/o	Melanesian
38	*IFIH1*	c.1528T>C	NM_022168.3	Missense	Heterozygous	Family history of CKD, nephropathy of unknown origin, ESKD at 44 y/o	Melanesian
47	*OCRL*<br>*OCRL*	c.40A>G<br>c.52A>G	NM_000276.3<br>NM_000276.3	Missense<br>Missense	Heterozygous<br>Heterozygous	Family history of CKD, nephropathy of unknown origin, ESKD at 46 y/o	Melansian
52	*NPHS1*	c.1321G>A	NM_004646.3	Missense	Homozygous	Family history of CKD, ESKD at 27 y/o	Melanesian
66	*CFI*<br>*PKHD1*	c.1015C>T<br>c.950_957dup	NM_000204.3<br>NM_138694.3	Nonsense<br>Frameshift	Heterozygous<br>Heterozygous	Family history of CKD, ESKD at 51 y/o	Melanesian

Supplementary Table S1: Molecular and clinical characteristics of patient with Variants of Undetermined Significance (VUS).

CKD: chronic kidney disease; ESKD: End stage kidney disease; y/o: years old
